# Exercise Training, Intermittent Fasting and Alkaline Supplementation as an Effective Strategy for Body Weight Loss: A 12-Week Placebo-Controlled Double-Blind Intervention with Overweight Subjects

**DOI:** 10.3390/life10050074

**Published:** 2020-05-21

**Authors:** Kuno Hottenrott, Tanja Werner, Laura Hottenrott, Till P. Meyer, Jürgen Vormann

**Affiliations:** 1 Institute for Performance Diagnostics and Health Promotion, Martin-Luther-University of Halle-Wittenberg, 06108 Halle, Germany; 2 NuOmix Research k.s. Applied Nutriomic Research, Martin, 81106 Bratislava, Slovaki; werner@nuomix-research.com; 3 Faculty of Sport Science, Ruhr-University Bochum, 44801 Bochum, Germany; laura.hottenrott@rub.de; 4 Institute of Sport Science, Martin-Luther-University of Halle-Wittenberg, 06108 Halle, Germany; till-meyer@gmx.net; 5 Institute for Prevention and Nutrition, 85737 Ismaning, Germany; vormann@ipev.de

**Keywords:** intermittent fasting, weight loss, running performance, acid–base, alkaline supplementation

## Abstract

Background: Intermittent fasting (IF) combined with exercise has been suggested to enhance weight loss. However, both procedures might negatively influence acid–base status. The aim of this study was to determine the combined effects of IF, exercise training and alkaline supplementation in overweight subjects on body composition and running performance. Methods: 80 overweight subjects of age 45.5 ± 7.8 years were assigned to IF or non-intermittent fasting (nIF). Furthermore, subjects were randomly assigned to take either an alkaline supplement (IF-v, nIF-v) or a placebo (IF-p, nIF-p) twice a day. All subjects performed a personalized endurance exercise program (3–4 times/week for 12 weeks). Body weight, body composition, running performance and acid–base parameters were determined before (pre) and after the 12-week program (post). Results: 68 participants completed the study. There was a significant effect on body weight loss, body fat loss, visceral fat loss and running performance enhancement in all groups (*p* < 0.01) for pre and post measurements. Body weight decreased in all groups (IF-p: −5.80 ± 0.77 kg and nIF-p: −3.40 ± 0.58 kg; IF-v: −8.28 ± 0.75 kg and nIF-v: −5.59 ± 0.87 kg). In both dietary strategies, weight loss was significantly further enhanced by alkaline supplementation. The increase in running velocity was significantly higher in IF combined with alkaline supplementation (IF-v 1.73 ± 0.23 km/h and IF-p 0.97 ± 0.20 km/h). In addition, alkaline supplementation increased plasma HCO_3_^−^ concentration and urinary pH. Conclusion: Exercise training in combination with IF and alkaline supplementation is an effective strategy to reduce body weight and improve running performance in a 12-week intervention.

## 1. Introduction

It is generally known that being overweight is a major risk factor for various diseases. Therefore, losing weight is a proven method to reduce overweight-associated risk factors [1] and mortality [2]. Generally, there are two strategies for weight loss: to increase physical activity and/or decrease caloric intake. To follow a calorie-restricted diet every day can be difficult for overweight individuals with poor long-term compliance. An alternative to a daily general reduction of caloric intake can be intermittent fasting (IF). The most popular scheme of IF includes two days with very low intake of calories per week (5:2 method). Compared to continuous energy restriction, IF has been shown to be similarly effective concerning weight loss while having even more beneficial effects on glucose and lipid homeostasis [3]. As individuals adhering to IF have to focus on energy intake only for defined days a week, compliance with this diet strategy is easier to achieve than with continuous energy restriction [4,5].

It is undisputed that exercise on its own has many beneficial health effects but a low efficacy for weight reduction. Therefore, it is reasonable to assume that the combination of IF with exercise training is more effective than a single strategy. The efficacy of combined exercise and diet interventions has already been shown in type 2 diabetes prevention [6]. Adherences to exercise interventions are higher when accompanied with measured performance improvements. All interventions with the aim of reducing body weight will result in increased fat mobilization. High rates of lipolysis, β-oxidation and CoA synthesis cause acidosis [7], resulting in ketone production leading to a transient reduction in buffering capacity. Biochemically, an acidic environment has been shown to reduce triglyceride release from adipocytes and as a consequence, the acidosis may lead to an inhibition of weight loss [8]. Furthermore, a transient reduction in buffering capacity could impair the ability to compensate for evolving acidosis during exercise with the result of limiting performance [9]. In athletes, various studies have shown that strategies improving buffering capacities resulted in better performance [10]. Alkaline supplementation may circumvent fasting-induced acidosis, leading to enhanced weight loss and improved performance.

To test this, healthy overweight individuals participated in a 12-week exercise program with or without IF. In addition to the exercise program and the dietary strategy, the influence of alkaline supplementation on weight loss, running performance and acid–base status was investigated.

## 2. Materials and Methods

### 2.1. Ethics

The study was approved by the Ethical Committee of the Martin-Luther-University, Halle-Wittenberg (Germany) (Number: 2015-04). Subjects gave their written informed consent according to the Declaration of Helsinki.

### 2.2. Subjects and Study Design

**Figure 1:**
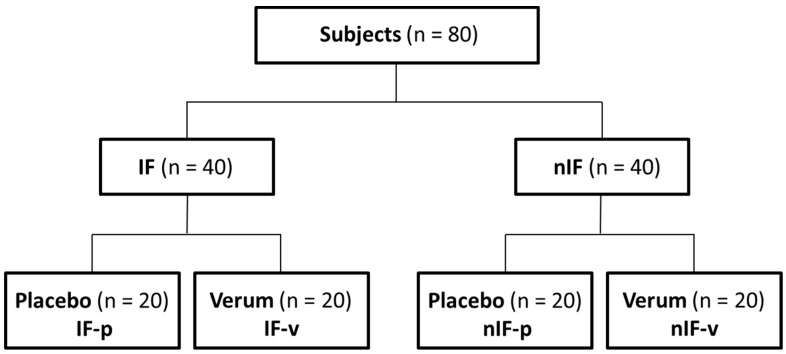
Study design. Intermittent fasting (IF); non-intermittent fasting (nIF).

Study subjects were recruited by newspaper advertisement. A total of 80 healthy individuals met the inclusion criteria regarding age (20–60 years), body mass index (BMI; 25–30 kg/m^2^) and physical activity (1–2 h exercise/week). A total of 40 men and 40 women were equally assigned to different dietary strategies: IF and nIF. Further on, in a double-blinded placebo-controlled design, subjects received either an alkaline mineral supplement (Basica Direkt^®^, Protina Pharmazeutische GmbH, Ismaning, Germany) or a similar tasting placebo (ingredients: citric acid, sorbitol, lemon flavor and calcium stearate) twice a day (morning and evening) (Figure 1). One stick of Basica Direkt^®^ contains 120 mg calcium, 200 mg magnesium, 2.5 mg zinc, 25 µg molybdenum, 20 µg chromium, 15 µg selenium. Within the study, all subjects followed an individual exercise program for 12 weeks. Body weight, body composition, running performance, dietary intake and acid–base parameters were measured before and after the 12-week intervention.

### 2.3. Exercise Program

All participants performed a personalized endurance training based on heart rate determined by an incremental lactate threshold field-test before the start of the 12-week program (incline in running velocity of 1.5 km/h every 800 m until exhaustion starting at an initial speed of 6 km/h for women and 7 km/h for men) Following the test results, subjects received training plans based on performance status and individual heart rate training zones. The 12-week exercise program consisted of 30–60 min running and 20 min of strength training 3 to 4 times a week. Additionally, all participants completed a walking program up to 2 h every weekend. The training was done individually and training plan adherence was monitored by protocol.

### 2.4. Dietary Strategy

Before the start of the 12-week intervention, after 6 weeks into the intervention and in the last week of the intervention, dietary intake of all subjects was monitored with standardized protocols for 7 days. At the start of intervention, to adjust to the fasting program, IF-participants started with half-day fasting (800/1200 kcal per day, women/men) three times a week for the first three weeks. After the initial adjusting period, the following IF program included two fasting days weekly with a daily caloric intake of 400 kcal for female and 600 kcal for male participants and 5 days of ‘normal’ caloric consumptions (covering the individual demand). Diet plans for fasting (half-)days were provided. Both nIF and IF participants received advice about a balanced diet in a two-hour seminar and a guidebook. Recommendations on a normal balanced diet were based on international guidelines on sports nutrition consisting of 50–60% carbohydrates, 25–30% fat and 15–20% protein of total daily calories [11]. All participants received recommendations on their individual daily caloric intake based on their basal metabolic rate and their activity level.

### 2.5. Test Parameters

Body weight, body composition and basal metabolic rate were measured with a Tanita^®^ BC-545N Segment Body analyzer (Tanita Europe BV, Amsterdam, Netherlands) under similar conditions in the pre and post test.

In the field test, the initial speed of 6 km/h for women and 7 km/h for men was increased by 1.5 km/h every 800 m until exhaustion to determine maximum running velocity and anaerobic threshold. During the test, heart rate was recorded with heart rate monitor RS 800CX (Polar, Kempele, Finland) continuously. Lactate was measured with the enzymatic-amperometric method (Dr. Mueller, Super GL ambulance, Freital, Germany) in a 10 μL blood sample taken from an ear lobe after each stage. Collected data were processed with the software WinLactat 3.1 (Mesics, Münster, Germany). The individual anaerobic threshold was derived from the lactate–velocity curve using the Dickhuth model [12].

Before and after the 12-week program acid–base parameters (pH_b_, pCO_2_), glucose, lactate and serum concentration of minerals (Na^+^ Mg^2+^, K^+^, Ca^2+^) were measured using a capillary blood sample of 200 µL taken from the hyperemized earlobe and immediately analyzed by a blood gas analyzer (phox^®^Plus M Analyzer, nova^®^biomedical, Cheshire, UK). Bicarbonate (HCO_3_^−^) was determined on the basis of the Henderson–Hasselbalch equation. Every morning, urinary pH (first urine of the day) was self-measured by participants with pH-sticks (Macherey-Nagel, Düren, Germany).

### 2.6. Statistics

Statistical analysis was performed with SPSS 22.0 (IBM, USA). Gaussian distribution of the data were tested with the Shapiro–Wilk Test. Two-way ANOVA and Student’s t-Test were used to test changes and group differences for weight loss, body fat, running performance, acid–base parameters and blood mineral concentrations. For all statistical tests, *p* < 0.05 was considered statistically significant. *p*-Values are provided and data are presented as mean (M) and standard error of mean (± SEM).

## 3. Results

During the 12-week study period, 12 participants dropped out (7 men, 5 women) because of injuries, illness or personal reasons or stopped taking the alkaline mineral supplement regularly. Altogether, there were 68 participants for statistical assessment (18 IF-p, 17 IF-v, 17 nIF-p, 16 nIF-v). There were no significant differences in baseline data for age and body composition. Exercise history was also similar between the verum (1.3 ± 1.1 running hours/week) and placebo groups (1.2 ± 1.4 running hours/week). Changes were calculated between baseline (start of the program) and after 12 weeks (end of the program). Before the start of the intervention, no differences in the distribution of carbohydrate, protein and fat intake between the verum and placebo groups were found. Basal metabolic rate decreased in all four groups from pre test (IF-p: 1767 ± 312; IF-v: 1755 ± 337; nIF-p: 1706 ± 274; nIF-v: 1743 ± 372) to post test (IF-p: 1674 ± 289; IF-v: 1727 ± 308; nIF-p: 1685 ± 266; nIF-v: 1697 ± 373) significantly. The three times of 7 days dietary monitoring showed no differences in acid load calculated on basis of PRAL scores (Potential Renal Acid Load) [13] and no differences between groups in the composition of carbohydrate, fat and protein content. There were no differences between the three dietary monitoring weeks (one week before: 52.3 ± 2.2% carbohydrates, 30.7 ± 1.7% fat 17.0 ± 2.1% protein, after 6 weeks: 50.9 ± 2.4% CHO, 31.3 ± 1.9% fat 18.8 ± 1.7% protein and in the last week: 49.4 ± 2.6% CHO, 33.1 ± 2,0% fat 17.5 ± 1.8% protein).

Overall, there was a significant effect of the 12-week training program on body weight loss, loss of body fat, loss of visceral fat as well as on running performance. There was a trend that the training program decreased bicarbonate concentration in blood plasma.

### 3.1. Weight Loss

**Figure 2:**
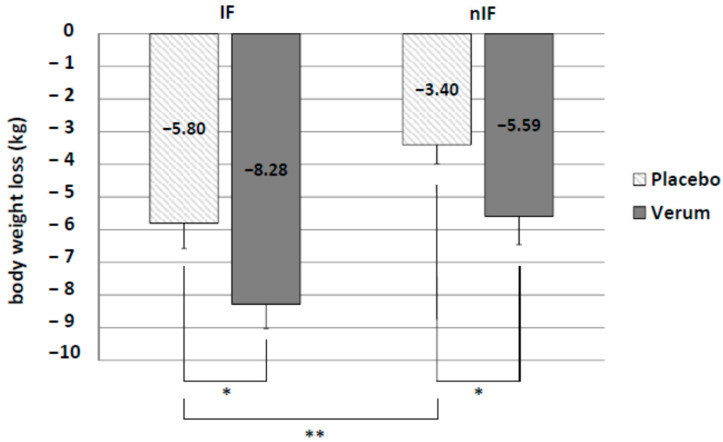
Significant differences in body weight loss in IF and nIF as well as placebo and alkaline supplementation group (verum). * *p* < 0.05; ** *p* < 0.01.

IF significantly increased body weight loss compared to nIF (IF-p: −5.80 ± 0.77 kg and nIF-p: −3.40 ± 0.58 kg, *p* = 0.010). Alkaline mineral supplementation in addition to IF resulted in the highest body weight loss (IF-v: −8.28 ± 0.75 kg). Difference to placebo group was significant (*p* = 0.015). Furthermore, there was a significant difference between verum and placebo in nIF (nIF-v: −5.59 ± 0.87 kg and nIF-p: −3.40 ± 0.58 kg; *p* = 0.023) (Figure 2).

### 3.2. Fat Mass

**Figure 3:**
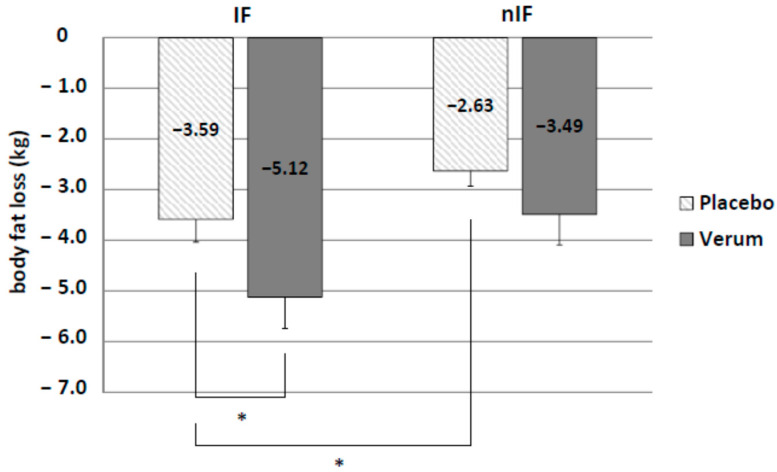
Significant differences in body fat loss in IF and additional alkaline supplementation (verum). * *p* < 0.05.

IF led to significantly higher fat mass loss than nIF (IF-p: −3.59 ± 0.45 kg and nIF-p: −2.63 ± 0.30 kg, *p* = 0.044). Additionally, there was a significant effect of the supplement on body fat loss in IF only (IF-v: −5.12 ± 0.62 kg and IF-p: −3.59 ± 0.45 kg; *p* = 0.028) (Figure 3).

### 3.3. Visceral Fat

**Figure 4:**
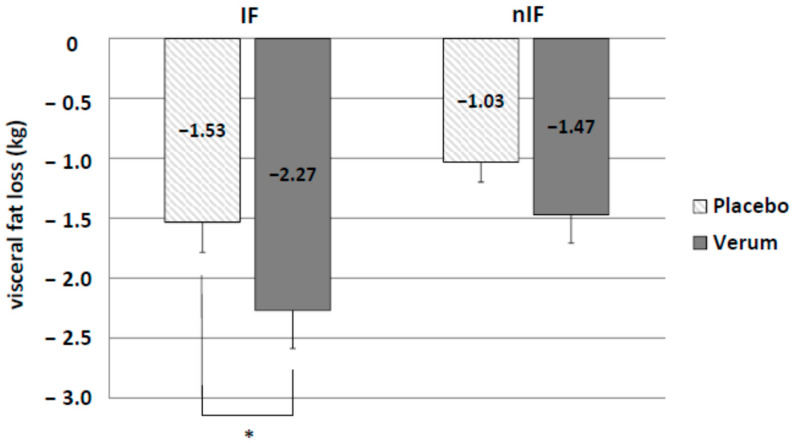
Significant differences in visceral fat loss by additional alkaline supplementation (verum) in IF only. * *p* < 0.05.

There was a clear trend towards enhanced loss of visceral fat by IF (IF-p: −1.53 ± 0.25 kg and nIF-p: −1.03 ± 0.17 kg) without reaching statistical significance (*p* = 0.054). Only supplementation in IF led to a significant higher loss of visceral fat (IF-v: −2.27 ± 0.37 kg and nIF-v: −1.53 ± 0.25 kg; *p* = 0.018) (Figure 4).

### 3.4. Running Performance

**Figure 5:**
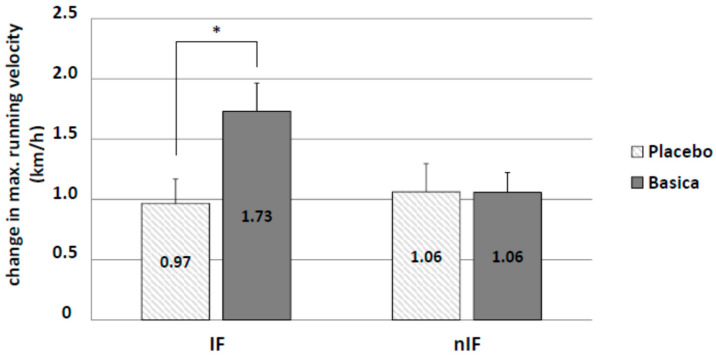
Significant difference in running velocity by additional alkaline supplementation (verum) from the start to the end of the intervention in IF only. * *p* < 0.05.

Improvement of running performance was determined by measuring changes in maximum running velocity. Overall, the 12-week training program increased running performance. IF had no additional effect. Only supplementation in IF led to a significant improvement in running velocity (IF-v: 1.73 ± 0.23 km/h and IF-p: 0.97 ± 0.20 km/h; *p* = 0.021) (Figure 5). There were no significant differences for lactate values at the individual anaerobic threshold between groups.

### 3.5. Acid–Base and Blood Parameters

**Figure 6:**
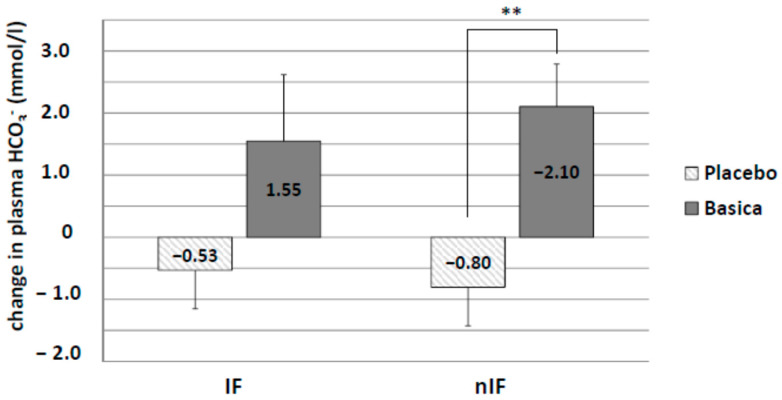
Changes in plasma bicarbonate concentration from the start to the end of the intervention. Alkaline supplementation group (verum) significantly differed in plasma bicarbonate in nIF only. ** *p* < 0.01.

Acid–base status was determined by measuring changes in plasma bicarbonate (HCO_3_^−^) levels as well as urinary pH. There was a decrease in plasma HCO_3_^−^ concentration in IF-p (−0.53 ± 0.53 mmol/L) and nIF-p (−0.80 ± 0.62 mmol/L) by the 12-week training program (Figure 6). Alkaline supplementation increased plasma HCO_3_^−^ concentration in both groups (IF-v: 1.55 ± 1.07 mmol/L and nIF-v: 2.10 ± 0.68 mmol/L), reaching statistical significance in nIF only (*p* = 0.0056).

**Figure 7:**
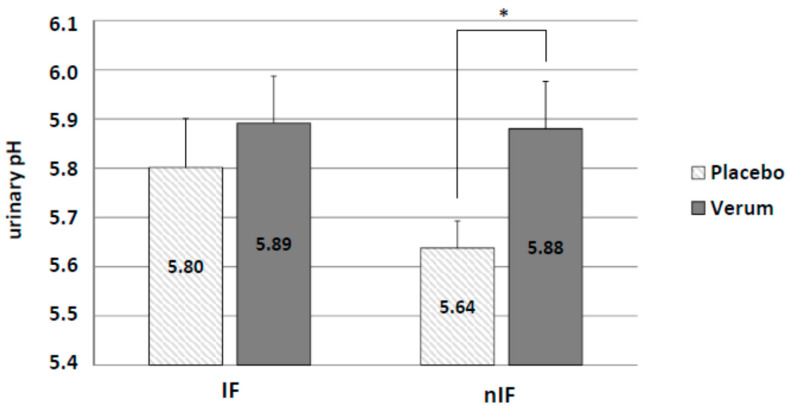
Alkaline supplementation group (verum) significantly differed in urinary pH from the start to the end of the intervention in nIF only. * *p* < 0.05.

Changes in urinary pH between the groups were assessed on the last day of intervention. Similar to the results for plasma HCO_3_^−^ concentration, alkaline supplementation increased urinary pH in IF (IF-p: 5.80 ± 0.10 and IF-v: 5.89 ± 0.10) and nIF (nIF-p: 5.64 ± 0.06 and nIF-v: 5.88 ± 0.10) with a significant effect (*p* = 0.038) in nIF only (Figure 7).

There were no significant differences in pH_b_, Glucose, Lactate, Na^+^, K^+^, Ca^2+^, Mg^2+^ between all groups.

## 4. Discussion

The exercise program alone is an effective weight loss strategy since all groups significantly lost body weight and fat. These effects were significantly enhanced by the dietary strategy of IF. The beneficial effects of the exercise program in fasting or non-fasting groups were further increased by alkaline supplementation, indicating that already the exercise program alone leads to a burden on acid–base balance that is further exaggerated by additional fasting. By supplying more alkaline minerals, the inhibitory effect increased acidosis can be neutralized in both groups.

Before the start of the intervention, dietary monitoring showed no differences in acid load between groups. This indicates that changes in acid–base status during the intervention are induced by the alkaline supplement.

Previous studies have shown that changes in acid–base status lead to hyperacidic conditions impairing the metabolism and release of triglycerides from adipose tissue [8]. Also, fasting is known to induce hyperacidic conditions [14] and decelerated fat metabolism [15]. This condition is the consequence of fasting-induced ketoacid production, which might limit the overall capacity to reduce body weight and fat content. However, Hood et al. [16] showed an increase in ketogenesis and ketoacid excretion by alkaline supplementation during fasting, which indicates increased mobilization of fat. This agrees with the results of the present study, also showing that alkaline mineral supplementation significantly increased fat loss in fasting subjects.

Alkaline supplementation increased plasma HCO_3_^−^ concentration and urinary pH, indicating a better capability to avoid local acidosis. As there were no differences in serum concentrations of Mg^2+^, Ca^2+^, K^+^ and Na^+^, the effect of the supplement is due to its alkalinity.

In all groups, the exercise program led to significant improvement in running performance. A further increase in running performance could only be seen in IF under alkaline supplementation. It is known that already after 5 min exercise, a local acidosis in muscle occurs [17]. This local acidosis might impair energy metabolism and finally, muscle performance. By alkaline supplementation, the work load-induced increase in local acid concentration could be avoided [18]. Similar to the effects observed with regard to weight loss, the mechanism of this performance enhancement can be explained by the alkaline supplementation reducing the local acidosis resulting from fasting and intense running. Increased local buffering capacity in combination with the benefits of fasting and endurance sport [19,20] enables a greater improvement in muscle performance.

Several major physiological responses to fasting are similar to those caused by regular aerobic exercise including increased insulin sensitivity, cellular stress resistance, reduced resting blood pressure and heart rate, and weight loss [21,22]. In particular, IF combined with exercise appears to have synergistic effects on energy metabolism due to mitochondrial biogenesis and fat metabolism [19,21,23], and on weight loss [24].

Generally, the achieved weight loss will significantly reduce the risk for overweight-associated health risks. Beneficial effects of IF have already been observed by others: Johnstone [25] described IF as a potential strategy for weight loss with good feasibility. Harvie et al. [4] concluded that IF is a safe, practical and long-lasting alternative method compared to conventional dieting. In our study, participants’ feedback confirmed that IF is a practical strategy to reduce caloric intake with the advantage to follow a normal caloric diet during non-fasting days.

### Limitation

We monitored nutritional intake three out of eight weeks only. Before the start of the 12-week intervention, after 6 weeks into the intervention and in the last week of the intervention, dietary intake of all subjects was monitored with standardized protocols for 7 days. There were no special recommendations on allowed and prohibited foods. Recommendations on a normal balanced diet were based on international guidelines on sports nutrition consisting of 50–60% carbohydrates, 25–30% fat and 15–20% protein of total daily calories [11]. Sampling of 24-h urine would be needed to measure ketone excretion, which was not possible in this population study. Use of keto-sticks for urinary detection of ketones as an indicator of fasting-induced acidosis in morning urine was not sensitive enough for quantitative evaluation. Further studies should use a more sensitive method to measure ketone excretion.

## 5. Conclusions

IF in combination with a 12-week exercise program was more effective in body weight and body fat reduction than an exercise program with a normal diet. In addition, alkaline supplementation supports optimal weight and fat loss in fasting as well as non-fasting subjects following an exercise program. The exercise program led to enhanced performance, which was further improved by alkaline supplementation in IF.
